# OPTIMIZING THE CLINICAL FUNCTIONING INFORMATION TOOL (ClinFIT) IN ROUTINE CLINICAL PRACTICE: DEVELOPMENT OF FUNCTIONAL STAGING CUTOFF SCORES FOR REHABILITATION PROVISION AND INTENSITY

**DOI:** 10.2340/jrm.v57.44170

**Published:** 2025-10-29

**Authors:** Bhasker AMATYA, Adrian MARTINEZ-DE LA TORRE, Masahiko MUKAINO, Krystal SONG, Melissa SELB, Gerold STUCKI, Fary KHAN

**Affiliations:** 1 Department of Rehabilitation Medicine, Royal Melbourne Hospital, Parkville, Victoria; 2 Department of Medicine (Royal Melbourne Hospital), University of Melbourne, Parkville, Victoria; 3 Australian Rehabilitation Research Centre, Royal Melbourne Hospital, Parkville, Victoria; 4 Peter MacCallum Cancer Center, Parkville, Victoria, Australia; 5 Faculty of Health Sciences and Medicine, University of Lucerne, Lucerne; 6 Swiss Paraplegic Research, Nottwil, Switzerland; 7 Department of Rehabilitation, Hokkaido University Hospital, Sapporo, Japan; 8 ICF Research Branch, Nottwil; 9 Center for Rehabilitation in Global Health Systems, University of Lucerne, Lucerne, Switzerland

**Keywords:** functioning, rehabilitation, ClinFIT, outcome measures

## Abstract

**Objective:**

To develop data-driven functional staging cutoff scores for the Clinical Functioning Information Tool (ClinFIT) total raw score to stratify patients according to rehabilitation provision and intensity.

**Methods:**

This observational study included adult inpatients (*n* = 270) admitted to a tertiary rehabilitation unit. ClinFIT total scores at admission were analysed alongside the Therapy Disciplines domain of the Rehabilitation Complexity Scale to represent rehabilitation intensity. Receiver Operating Characteristic analysis was used to identify optimal cutoff points distinguishing between levels of rehabilitation intensity. Subgroup analyses were conducted by age, sex, and diagnosis.

**Results:**

Participants were predominantly male (54.1%), with a mean age of 62.9 ± 14.3 years. ClinFIT total raw scores improved significantly across all health conditions at discharge compared with admission (*p* < 0.001), reflecting substantial functional recovery during inpatient rehabilitation. Two ClinFIT total score cutoffs were identified: 135 (light vs moderate) and 192 (moderate vs high intensity), with acceptable discriminatory performance (AUCs: 0.720, 0.748, respectively). Subgroup analyses supported the robustness of this 3-level staging system across demographic and diagnostic groups.

**Conclusion:**

This study provides evidence-based cutoff scores for ClinFIT, supporting its clinical use for stratifying rehabilitation provision and intensity. These findings may enhance clinical decision-making, optimize resource allocation, and promote wider adoption of the ClinFIT. Further validation in external and diverse populations is warranted.

## LAY ABSTRACT

Clinical Functioning Information Tool (ClinFIT) is an instrument for clinicians to assess how health conditions affect a person’s ability to function in daily life. It provides a total raw score of 0–300, higher scores indicate greater functional impairment. This study developed cutoff scores for the ClinFIT to guide the level of rehabilitation support required for a patient in everyday rehabilitation practice. ClinFIT data from 270 adults in a rehabilitation unit were analysed at admission and discharge. Two key cutoff scores (135 and 192) were identified, dividing patients into light, moderate, and high rehabilitation intensity groups. These categories reflected differences in therapy needs and progress. This can guide clinical decision-making, help plan therapy intensity, and improve use of rehabilitation resources. This functional staging system can support more consistent and equitable rehabilitation care across different patient groups and settings.

Functioning represents a dynamic interaction between health conditions, body function and structure impairments, activity limitations, participation restrictions, and contextual factors (1). Poor functional capacity is associated with adverse outcomes such as increased complications, prolonged hospital stays, and higher mortality rates (2, 3). Accurate and standardized assessment of patients’ functional status is essential in routine practice for clinical decision-making, to optimize treatment strategies, improve patient health, and reduce healthcare burden management (4, 5). Despite its importance, there are significant gaps in patient assessment across the continuum of care, particularly in rehabilitation settings (6).

The introduction of the Clinical Functioning Information Tool (ClinFIT) by the International Society of Physical and Rehabilitation Medicine (ISPRM) has created a significant opportunity to enhance the assessment of functional outcomes in clinical and rehabilitation settings (7, 8). ClinFIT addresses a critical gap in evaluating and monitoring functional status by providing standardized, comprehensive data on patient functioning (7). The tool (previously referred to as the Rehabilitation Set or ICF Generic-30 Set with simple descriptions) is scientifically robust and can contribute to optimal rehabilitation care in clinical practice, clinical quality management, and research (9–11). ClinFIT has demonstrated its feasibility in various clinical settings, highlighting its potential to track functional changes over time and enhance clinical decision-making (6, 10–12). A scoping review comparing ClinFIT with 12 commonly used multidimensional rehabilitation outcome measures showed that ClinFIT demonstrated broader coverage of rehabilitation-relevant concepts compared with these tools (13). Further, this study underscores its potential to unify functional assessment in rehabilitation, enabling consistent data collection across diverse clinical and research settings. Despite its potential, ClinFIT has not been widely adopted in routine clinical practice and is primarily utilized for research purposes (13). Many clinicians continue to rely on traditional or subjective evaluation methods due to familiarity, perceived complexity of new tools, or lack of established meaningful clinical utility scores for implementation (13).

A promising strategy to enhance the clinical utility of ClinFIT is to develop cutoff scores that identify a patient’s clinically meaningful functional stage at specific points along the continuum of care. This, in turn, may support decision-making and monitoring in rehabilitation (14–17). By stratifying patients according to their functional abilities, clinicians can develop individualized treatment plans that address specific needs and goals, which ensure that care is tailored to each patient’s unique circumstances, leading to improved outcomes and more effective rehabilitation strategies. Additionally, functional staging facilitates the prioritization of healthcare resources, as, based on the patient’s needs, clinicians can allocate staff time, therapy sessions, and specialized equipment more efficiently, ensuring equitable access to care. Furthermore, categorizing patients by functional level enables clinicians to evaluate and monitor progress systematically. Patterns of improvement, deterioration, or stagnation of outcomes can be identified early, allowing for timely adjustments to treatment plans and more precise goal setting. Functional staging can also promote interdisciplinary collaboration in rehabilitation settings by ensuring that all members of the interdisciplinary team (rehabilitation physicians, allied health professionals, nurses, social workers, and others) are aligned in their treatment approach. This enhances the consistency and quality of care delivered across disciplines and provides a framework for predicting recovery trajectories and setting realistic expectations for patients and their families. Moreover, understanding a patient’s functional stage allows clinicians to communicate likely outcomes effectively and plan long-term care strategies accordingly.

ClinFIT assessment currently generates a total raw score ranging from 0 to 300, with higher scores indicating greater functional impairment. This total score alone does not allow for effective staging. Establishing meaningful cutoff points for the total raw score, i.e., indicating the threshold where one functional stage ends and another begins, is vital to facilitate standardized and more precise functional grading. More precise functional staging is expected to enhance clinical decision-making and optimize rehabilitation planning and outcome evaluation. To our knowledge, no previous studies have attempted to establish cutoff scores for ClinFIT, highlighting a critical gap in its clinical utility. The aim of this study was to define data-driven functional staging cutoff scores for the ClinFIT total raw score to stratify patients according to rehabilitation provision and intensity. The study also aimed to evaluate the consistency of these cutoff scores across key demographic and diagnostic subgroups.

## METHODS

### Setting and participants

This observational pilot study was a part of a quality improvement initiative at the 40-bed medically supervised inpatient rehabilitation unit of the Royal Melbourne Hospital (RMH), a tertiary referral centre in Victoria, Australia. This study was performed in accordance with the Strengthening the Reporting of Observational Studies in Epidemiology (STROBE) reporting guidelines (18) and was approved by the hospital Ethics Committee (HREC no. QA2025049). All patients admitted to the rehabilitation unit in the last 4 years (from January 2021 to December 2024) with various health conditions, including neurological, musculoskeletal, cancer, and other disabling conditions, were eligible for inclusion. All patients aged 18 years and above, admitted to the rehabilitation unit with complete ClinFIT data on both admission and discharge, were included. All participants received individualized, interdisciplinary rehabilitation tailored to their clinical needs.

### Procedure and data collection

As per routine clinical practice, all patients admitted to the rehabilitation ward underwent assessment on admission (T0) and discharge (T1) by the interdisciplinary team. The data were retrospectively sourced from the electronic medical records (EMR) over the past 4 years (2021 to 2024) and previously completed ClinFIT-related projects within the department. Data were extracted and entered into the project database by an independent research officer, a clinician with prior training and experience in using ClinFIT. Data collected included demographic information (e.g., age, sex, marital status, education, and employment), disease-related details (e.g., primary diagnosis, date of diagnosis, symptoms, and medications), and clinical parameters such as length of stay (LOS), rehabilitation interventions received (e.g., physical, speech, and occupational therapy, social worker, dietary guidance, etc.), and other outcomes (e.g., discharge destination, referral patterns, and presence of complications). The number and type of therapy disciplines involved (hereafter referred to as “therapy involvement”) were recorded from interdisciplinary team notes/discharge summaries and EMR-based referral logs. Therapy involvement was summarized as the cumulative count of distinct disciplines involved on discharge. All data were de-identified and checked for completeness; cases with missing primary outcome data were excluded. No imputation was performed.

### Measures

The *Clinical Functioning Information Tool* (*ClinFIT*) (7, 8) comprises 30 categories divided into 2 domains: 9 items under “Body Functions” and 21 items under “Activities and Participation”. Each category includes clinically meaningful descriptions, enabling clinicians to assess patients’ functional problems using an 11-point numeric rating scale, where 0 indicates no problem and 10 represents a complete problem, resulting in a total score range of 0–300, with higher scores indicating greater functional limitation. The total ClinFIT score on admission (T0) was used as the primary functional measure.

### Statistical methods

*Descriptive analysis.* Descriptive statistics were used to summarize patient characteristics and ClinFIT scores at both time points (T0 and T1). Changes in ClinFIT scores between admission and discharge were assessed using paired *t*-tests. Effect size statistics were calculated and assessed against Cohen’s criteria (0.2 as small, 0.5 as medium, and 0.8 as large effect) (19, 20). A *p*-value of ≤ 0.05 was considered statistically significant. All analyses were done in R statistical software version 4.3.1 (R Foundation for Statistical Computing, Vienna, Austria).

*Rehabilitation intensity categorization.* Rehabilitation intensity was defined using the Therapy Disciplines (TD) domain of the Rehabilitation Complexity Scale version 2 (RCS v2), a validated ordinal measure that captures the number of therapy disciplines actively involved in a patient’s care (21). This domain serves as a proxy for the complexity and intensity of rehabilitation services. In the RCS v2 framework, patients are categorized into 4 levels: TD0: no therapist involvement; TD1: 1 discipline; TD2: 2 to 3 disciplines; and TD3: 4 or more disciplines (21). Although originally developed in the context of neurorehabilitation, RCS v2 has also been applied in other populations, including trauma patients (21, 22). Furthermore, extended versions of the RCS have been validated across a broader range of diagnostic groups (23). The RCS-TD levels were used as the reference for categorizing rehabilitation intensity. The TD domain therefore captures the intensity of rehabilitation provision (delivered services) rather than directly assessing needs.

*Cutoff point identification.* To identify ClinFIT total score thresholds that best distinguish between levels of rehabilitation intensity, we applied a Receiver Operating Characteristic (ROC) based procedure (24). The ClinFIT total score, ranging from 0 to 300, was used as the continuous predictor, and the outcome variable was defined according to the RCS v2 Therapy Disciplines classification. For each pair of adjacent levels, a binary outcome was constructed to compare one intensity level against the next. For every possible ClinFIT cutoff value, we calculated standard diagnostic performance metrics, including sensitivity, specificity, Youden Index (sensitivity + specificity − 1), accuracy, and the area under the ROC curve (AUC) (25). The optimal cutoff for each pairwise comparison was defined as the value that maximized the Youden Index, reflecting the best balance between sensitivity and specificity. After determining the optimal threshold for a given comparison, individuals in the higher-intensity group were excluded, and the process was repeated for the remaining categories.

This stepwise procedure was applied until all adjacent category comparisons were evaluated. The number and location of cutoff points were not predefined but were determined empirically based on the number of outcome categories and the discriminative capacity of the ClinFIT score. This allowed for a flexible and data-driven classification approach that remained grounded in the structure of the RCS v2 and the empirical distribution of ClinFIT scores in each sample or subgroup.

Following the initial ROC analysis, adjacent outcome categories were further assessed for potential merging based on the proximity of their associated cutoff values and overall performance. Specifically, categories were collapsed if the resulting ClinFIT cutoffs were separated by less than 5% of the total scale range (i.e., fewer than 15 points on the 0–300 scale) or if the discriminatory power between them was limited (defined as an AUC below 0.6 or a Youden Index below 0.3). This step ensured that the final classification retained only distinctions that were both clinically interpretable and statistically meaningful. The final number of rehabilitation intensity categories was determined empirically, based on data distribution, ROC performance, and clinical plausibility, to produce a robust and interpretable functional staging system.

*Subgroup analyses.* To explore the potential variability in ClinFIT-based rehabilitation staging across different patient populations, we conducted subgroup analyses stratified by demographic and clinical characteristics. Subgroups were defined by sex (male, female) and age group (under 65 years, 65 years or older, a commonly used cutoff in rehabilitation research to distinguish working-age from older adults) (26). Additional analyses were performed by primary diagnosis on admission, categorized into stroke, musculoskeletal (MSK) conditions, cancer, and other diagnoses. Within each subgroup, we independently applied the primary procedure to assess whether ClinFIT cutoffs varied across demographic and diagnostic groups, collapsing RCS v2 Therapy Disciplines categories per the primary criteria (cutoff separation < 5% of the scale, or weak discrimination: AUC < 0.6 or Youden < 0.3), generating ROC-based cutoffs between adjacent levels, and determining the number and location of cutoff points based on predefined criteria. As a result, the number of cutoff points and classification levels could vary across subgroups, depending on the distribution of rehabilitation intensity and the discriminatory performance of the ClinFIT score within each group. This approach enabled the development of subgroup-specific functional staging systems, tailored to the characteristics of each population.

## RESULTS

### Sociodemographic and clinical characteristics

A total of 270 patients were included in the study. Participants were predominantly male (*n* = 146, 54.1%), with a mean age of 62.9 years (SD = 14.3), with 49.6% of participants aged 65 years or older. The most frequent admission diagnosis was stroke (33.0%), followed by cancer (23.3%), musculoskeletal conditions (21.1%), and other diagnoses (22.6%). Common comorbidities included hypertension (40.4%), diabetes (6.3%), and depression (4.1%), while 16.7% of patients reported no comorbidities on admission. Most patients were discharged home (85.2%), with smaller proportions discharged to residential care (6.7%), acute hospital (4.1%), or other destinations (4.1%). Regarding therapy involvement on discharge, 34.4% of patients were classified as TD0 (no referrals), 36.3% as TD1 (referral to 1 discipline), 23.7% as TD2 (referrals to 2 or 3 disciplines), and 5.6% as TD3 (referrals to 4 or more disciplines), based on the RCS v2 classification. Most patients lived with a partner/family (62.2%) and 34.4% lived alone. Educational attainment was mainly secondary (52.6%), with 22.6% tertiary. On admission, 26.3% were working, 64.8% were unemployed or retired, and 7.0% were not working due to disability (Table I).

**Table I:** Participants’ demographic and clinical characteristics

Characteristics	Overall	Male	Female
Total, *n* (%)	270 (100%)	146 (54.1)	124 (45.9)
Age, years, mean (SD)	62.9 (14.3)	61.8 (14.9)	64.4 (13.3)
Above 65 years	134 (49.6)	71 (48.6)	63 (50.8)
Currently living with, *n* (%)			
Alone	93 (34.4)	52 (35.6)	41 (33.1)
Partner/Family	168 (62.2)	87 (59.6)	81 (65.3)
Other	9 (3.3)	7 (4.8)	2 (1.6)
Education level, *n* (%)			
Primary	32 (11.9)	15 (10.3)	17 (13.7)
Secondary	142 (52.6)	81 (55.5)	61 (49.2)
Tertiary	61 (22.6)	32 (21.9)	29 (23.4)
Not available	35 (13.0)	18 (12.3)	17 (13.7)
Employment status, *n* (%)			
Working	71 (26.3)	45 (30.8)	26 (21.0)
Unemployed or retired	175 (64.8)	91 (62.3)	84 (67.7)
Not working due to disability	19 (7.0)	10 (6.8)	9 (7.3)
Other	4 (1.5)	0 (0.0)	4 (3.2)
Not available	1 (0.4)	0 (0.0)	1 (0.8)
Admission diagnosis, *n* (%)			
Stroke	89 (33.0)	42 (28.8)	47 (37.9)
Musculoskeletal conditions	57 (21.1)	32 (21.9)	25 (20.2)
Cancer	63 (23.3)	36 (24.7)	27 (21.8)
Other	61 (22.6)	36 (24.7)	25 (20.2)
Comorbidities on admission, *n* (%)			
Hypertension	109 (40.4)	58 (39.7)	51 (41.1)
Diabetes	17 (6.3)	7 (4.8)	10 (8.1)
Depression	11 (4.1)	0 (0.0)	11 (8.9)
Ischaemic heart disease	4 (1.5)	2 (1.4)	2 (1.6)
None	45 (16.7)	23 (15.8)	22 (17.7)
Discharge destination, *n* (%)			
Home	230 (85.2)	124 (84.9)	106 (85.5)
Hospital	11 (4.1)	4 (2.7)	7 (5.6)
Residential	18 (6.7)	11 (7.5)	7 (5.6)
Other	11 (4.1)	7 (4.8)	4 (3.2)
Therapy disciplines involved on discharge (RCS v2 TD categories), *n* (%)			
TD0 (0 Referrals)	93 (34.4)	49 (33.6)	44 (35.5)
TD1 (1 Referral)	98 (36.3)	60 (41.1)	38 (30.6)
TD2 (2–3 Referrals)	64 (23.7)	29 (19.9)	35 (28.2)
TD3 (≥ 4 Referrals)	15 (5.6)	8 (5.5)	7 (5.6)
ClinFIT on admission, mean (SD)	165.3 (59.4)	163.6 (58.7)	167.2 (60.4)
ClinFIT on discharge, mean (SD)	97.5 (57.8)	93.6 (56.5)	102.2 (59.2)

ClinFIT: Clinical Functioning Information Tool; *n*: total number; RCS v2: Rehabilitation Complexity Scale version 2; SD: standard deviation; TD: therapy disciplines.

The mean ClinFIT score on admission was 165.3 (SD = 59.4), indicating moderate to severe functional impairment. On discharge, the mean ClinFIT score had improved to 97.5 (SD = 57.8), reflecting substantial functional recovery during inpatient rehabilitation. This improvement was observed across both sexes, with slightly lower discharge scores in males (93.6) compared with females (102.2). The difference between admission and discharge ClinFIT scores was statistically significant (*p* < 0.001), with a corresponding Cohen’s *d* effect size of 1.18 (95% CI: 1.04–1.32), indicating a large effect size for functional improvement over the course of inpatient rehabilitation.

### ClinFIT cutoff points for rehabilitation intensity

**Fig. 1:**
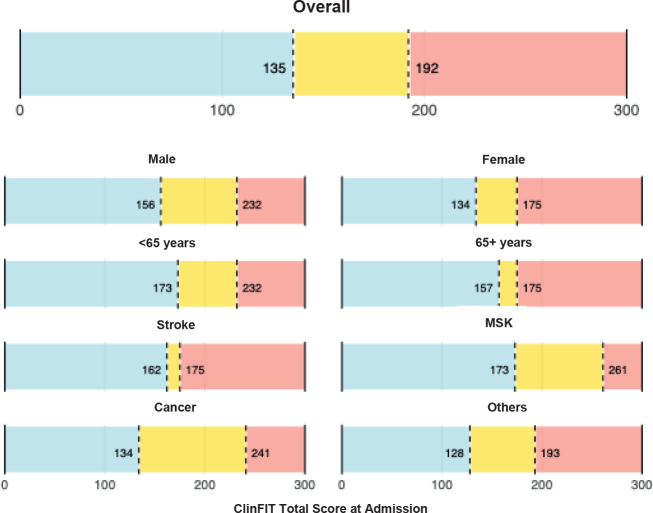
ClinFIT cutoff points: overall and stratified by demographics and diagnosis on admission. ClinFIT: Clinical Functioning Information Tool, MSK: musculoskeletal conditions.

ROC analyses were conducted to identify ClinFIT total score thresholds associated with rehabilitation intensity, using the RCS v2 Therapy Disciplines domain as the reference. Thresholds were retained if they demonstrated acceptable performance (AUC ≥ 0.6). Performance was evaluated using the area under the curve (AUC), sensitivity (Se), and specificity (Sp). Using this approach,2 clinically meaningful cutoff scores were identified from the ClinFIT total score on admission to stratify patients into 3 levels of rehabilitation intensity (Fig. 1). The first cutoff at 192 optimally discriminated between patients receiving high-intensity rehabilitation and those receiving moderate intensity, yielding a sensitivity of 0.8, specificity of 0.635, and an area under the ROC curve (AUC) of 0.748. The second cutoff at 135 separated moderate from light rehabilitation provision and intensity, achieving a sensitivity of 0.826, specificity of 0.556, and AUC of 0.72 (Table II). The corresponding Youden indices were 0.435 and 0.382, respectively. Further details on the procedure can be found in Table II. When applied to the full sample, these cutoff points classified 83 patients (30.7%) as receiving light-intensity rehabilitation, 82 patients (30.4%) as moderate-intensity, and 105 patients (38.9%) as high-intensity. This distribution reflects a balanced and interpretable staging system, with ClinFIT scores of ≤ 135 corresponding to light, 136–191 to moderate, and ≥ 192 to high rehabilitation intensity (Fig. 1).

**Table II:** Performance of ClinFIT total score cutoff values for classifying rehabilitation intensity on admission

Cutoff category	Cutoff	TP	FP	TN	FN	Se	Sp	Acc	AUC	Youden
High vs moderate	192	12	93	162	3	0.800	0.635	0.644	0.748	0.435
Moderate vs light	135	19	63	79	4	0.826	0.556	0.594	0.720	0.382

Note: The second classification step was conducted on the remaining sample after excluding patients classified as high intensity (ClinFIT ≥ 192) in the first step. As a result, the total sample size used to derive each cutoff differs, and the confusion matrix values do not sum to the full study population.

Acc: accuracy; AUC: area under the ROC curve, ClinFIT: Clinical Functioning Information Tool, FN: false negatives, FP: false positives, Se: sensitivity, Sp: specificity, TN: true negatives, TP: true positives.

### Subgroup analyses

The overall sample identified 2 thresholds: 135 (AUC: 0.720; Se: 0.826; Sp: 0.556) and 192 (AUC: 0.748; Se: 0.800; Sp: 0.635), supporting a 3-level functional staging model. A similar 3-stage structure was observed across sex and age groups. In males, thresholds at 156 (AUC: 0.683; Se: 0.783; Sp: 0.551) and 232 (AUC: 0.699; Se: 0.500; Sp: 0.913) were selected based on ROC performance. In females, 134 (AUC: 0.741; Se: 1.000; Sp: 0.607) and 175 (AUC: 0.800; Se: 1.000; Sp: 0.530) showed excellent sensitivity and consistent discrimination. Among participants under 65 years, cutoffs at 173 (AUC: 0.725; Se: 0.696; Sp: 0.719) and 232 (AUC: 0.947; Se: 1.000; Sp: 0.902) performed well. In those aged 65 or older, thresholds at 157 (AUC: 0.702; Se: 0.625; Sp: 0.842) and 175 (AUC: 0.637; Se: 0.818; Sp: 0.512) were identified.

Diagnostic subgroup results showed greater variability. Among stroke patients, 162 (AUC: 0.938; Se: 1.000; Sp: 0.917) and 175 (AUC: 0.724; Se: 1.000; Sp: 0.475) yielded strong discrimination. In musculoskeletal cases, thresholds at 173 (AUC: 0.637; Se: 0.722; Sp: 0.711) and 261 (AUC: 0.664; Se: 0.500; Sp: 1.000) were retained, though the latter was based on a small sample and should be interpreted with caution. In cancer patients, thresholds at 134 (AUC: 0.602; Se: 1.000; Sp: 0.365) and 241 (AUC: 0.951; Se: 1.000; Sp: 0.934) were retained. In patients with other conditions, although the threshold at 167 met statistical criteria (AUC: 0.708; Se: 0.429; Sp: 0.941), it was excluded due to limited sensitivity. Instead, 128 (AUC: 0.628; Se: 0.750; Sp: 0.500) and 193 (AUC: 0.644; Se: 0.500; Sp: 0.678) were retained for a more balanced and interpretable classification. ClinFIT cutoff points stratified by demographics and diagnosis on admission are presented in Fig. 1.

Overall, the ClinFIT total score showed good discriminatory capacity across subgroups. Although the exact thresholds varied, a consistent 3-stage structure emerged in most groups. However, in several subgroups, the number of patients classified within certain rehabilitation intensity levels, particularly the high-intensity group, was relatively small. As such, the corresponding thresholds should be interpreted with caution and viewed as exploratory. The full diagnostic performance results, including true and false positives and negatives (TP, FP, TN, FN), accuracy, and the Youden Index, are presented in Table III and Table IV.

**Table III:** Performance of ClinFIT total score cutoff values for classifying rehabilitation intensity on admission, stratified by sex and age

Cutoff category	Cutoff	TP	FP	TN	FN	Se	Sp	Acc	AUC	Youden
Male										
High vs moderate	232	4	12	126	4	0.500	0.913	0.890	0.699	0.413
Moderate vs llight	156	18	48	59	5	0.783	0.551	0.592	0.683	0.334
Female										
High vs moderate	175	7	55	62	0	1.000	0.530	0.556	0.800	0.530
Moderate vs light	134	6	22	34	0	1.000	0.607	0.645	0.741	0.607
< 65 years										
High vs moderate	232	4	13	119	0	1.000	0.902	0.904	0.947	0.902
Moderate vs light	173	16	27	69	7	0.696	0.719	0.714	0.725	0.414
≥ 65 years										
High vs moderate	175	9	60	63	2	0.818	0.512	0.537	0.637	0.330
Moderate vs light	157	5	9	48	3	0.625	0.842	0.815	0.702	0.467

Note: The second classification step was conducted on the remaining sample after excluding patients classified as high intensity (ClinFIT ≥ 192) in the first step. As a result, the total sample size used to derive each cutoff differs, and the confusion matrix values do not sum to the full study population.

Acc: accuracy; AUC: area under the ROC curve, ClinFIT: Clinical Functioning Information Tool, FN: false negatives, FP: false positives, Se: sensitivity, Sp: specificity, TN: true negatives, TP: true positives.

**Table IV:** Performance of ClinFIT total score cutoff values for classifying rehabilitation intensity on admission, stratified by diagnosis

Cutoff category	Cutoff	TP	FP	TN	FN	Se	Sp	Acc	AUC	Youden
Stroke										
High vs moderate	175	9	42	38	0	1.000	0.475	0.528	0.724	0.475
Moderate vs light	162	2	3	33	0	1.000	0.917	0.921	0.938	0.917
Musculoskeletal										
High vs moderate	261	1	0	55	1	0.500	1.000	0.982	0.664	0.500
Moderate vs light	173	13	11	27	5	0.722	0.711	0.714	0.637	0.433
Cancer										
High vs moderate	241	2	4	57	0	1.000	0.934	0.937	0.951	0.934
Moderate vs light	134	5	33	19	0	1.000	0.365	0.421	0.602	0.365
Other										
High vs moderate	193	1	19	40	1	0.500	0.678	0.672	0.644	0.178
Moderate vs light	128	12	10	10	4	0.750	0.500	0.611	0.628	0.250

Note: The second classification step was conducted on the remaining sample after excluding patients classified as high intensity (ClinFIT ≥ 192) in the first step. As a result, the total sample size used to derive each cutoff differs, and the confusion matrix values do not sum to the full study population.

Acc: accuracy; AUC: area under the ROC curve, ClinFIT: Clinical Functioning Information Tool, FN: false negatives, FP: false positives, Se: sensitivity, Sp: specificity, TN: true negatives, TP: true positives.

## DISCUSSION

This exploratory pilot study is the first to establish data-driven cutoff scores for the ClinFIT total score to stratify patients based on rehabilitation provision and intensity. We used the Therapy Disciplines domain of the RCS v2 as a proxy for rehabilitation intensity. Using a stepwise ROC-based procedure, we derived cutoff points across the full sample and a range of demographic and diagnostic subgroups. The findings support the feasibility of developing a ClinFIT-based functional staging model that reflects rehabilitation intensity with reasonable accuracy and potential for clinical application.

In the overall sample, 2 cutoff points, 135 and 192, demonstrated acceptable discriminatory performance and formed the basis of a 3-level functional staging system. These thresholds are consistent with established functional staging frameworks in rehabilitation (16, 17). The threshold at 192 was particularly helpful in identifying patients receiving high-intensity rehabilitation, while 135 effectively distinguished those with light vs moderate therapy involvement. These staging levels are not prescriptive but exploratory, intended to support clinical decision-making and resource allocation. Clinicians may use them as an adjunct to guide triage, anticipate service requirements, and benchmark patient groups, but individualized care planning remains essential.

The 2-threshold, 3-stage system was generally consistent across subgroups. Among both men and women, and across age categories, the retained thresholds varied in value but followed the same 3-stage structure. In men, thresholds at 156 and 232 were identified, and in women, 134 and 175 were retained. A similar pattern was observed in younger and older adults. Although the exact threshold values varied, the same 3-stage structure was consistently preserved. The frequent retention of a lower threshold between 130 and 135 reinforces its clinical relevance in separating light from moderate rehabilitation provision and intensity. While the 192 cutoff was not retained in every subgroup, its recurrence in the overall sample and multiple subgroup models supports its use as a reference point for distinguishing moderate from high intensity, without requiring strict adherence to that specific value across all populations. In clinical settings, the overarching thresholds identified in the full sample (135 and 192) may serve as a general guide when subgroup-specific thresholds are not available or when patient characteristics span multiple categories, such as age, sex, and diagnosis. We chose the RCS v2 Therapy Disciplines domain because it is routinely documented, standardizes the count of delivered disciplines, and has good measurement properties across settings, providing a reproducible proxy for rehabilitation provision and intensity (21–23).

Our findings align with the growing interest in using standardized functioning assessments to guide clinical decision-making and resource allocation in rehabilitation settings. This study adds to the growing evidence supporting ClinFIT’s clinical applicability by providing functional staging thresholds and expanding upon their conceptual scope. In comparison with existing functional assessment tools such as the Functional Independence Measure (FIM) (27) and the WHO Disability Assessment Schedule (WHODAS 2.0) (28), the ClinFIT offers several distinct advantages, particularly in terms of its comprehensive coverage and alignment with the ICF (13). Although FIM remains widely used in rehabilitation settings due to established cutoff scores for discharge planning and outcome prediction (29), its primary emphasis is on physical and cognitive function, with limited consideration of social participation and environmental factors (27). Further, it is associated with costs and requires specific training. The ICF framework-based WHODAS 2.0, though validated across diverse populations, is predominantly designed for self-report and may not fully capture clinical changes during intensive rehabilitation (28, 30). ClinFIT, by contrast, was designed for interdisciplinary clinician assessment and captures functioning across body functions, activities, and participation domains and is specifically designed for interdisciplinary clinician assessment (13, 31). This broader coverage offers a more holistic and context-sensitive measure of functioning and enhances its potential as a standardized tool for outcome measurement, clinical decision-making, and resource allocation in diverse rehabilitation populations (13). Further, ClinFIT is a freely available, non-proprietary assessment tool that holds significant potential for widespread global adoption, particularly in resource-limited settings.

### Strengths and limitations

This study has several strengths, including a clearly defined population, a standardized and clinically grounded outcome measure, and a rigorous, transparent analytic strategy. Stratified analyses by age, sex, and diagnosis enhance the generalizability of findings to varied patient populations. Nonetheless, several limitations must be acknowledged. First, its retrospective design may introduce selection or documentation bias. While ClinFIT was used as part of routine clinical practice in our unit, it is possible that some patients were not assessed. Second, the study was conducted in a single tertiary rehabilitation centre, which may affect the generalizability of the findings to other healthcare settings, particularly in low-resource or community-based environments. Third, the definition of rehabilitation intensity was based on referral patterns, which may not fully capture the complexity, duration, or quality of rehabilitation services delivered, and reflects actual provision rather than directly assessing patient needs. Further, the use of ClinFIT total scores as the primary metric, without accounting for potential domain-specific contributions (e.g., body functions vs activities and participation), may overlook important functional nuances (32). We acknowledge that alternative outcomes, such as functional change or prognosis, may be more appropriate for other clinical purposes, and that future research should explore staging cutoffs for these additional applications. Additionally, alternative staging approaches (e.g., ordinal regression, decision-tree methods, item response theory) may offer complementary insights and should be considered in future work. Lastly, the absence of an external validation cohort limits the robustness of the proposed cutoff scores. In several patient subgroups, particularly within the high-intensity rehabilitation category, the sample size was relatively small. In some instances, specific classification steps were based on a limited number of positive cases, which may compromise the statistical stability and reliability of the derived ClinFIT cutoff scores. Further, we excluded patients with missing discharge ClinFIT data to ensure data completeness and consistency, as some subgroup analyses compared admission and discharge scores. This could have reduced the sample size for staging analyses. These thresholds should therefore be interpreted with caution, as they may not be generalizable across broader clinical populations. As such, these preliminary thresholds should be considered exploratory until they are validated through replication in larger, more diverse, and independent patient cohorts. Future prospective multicentre studies are needed to confirm these findings and assess their applicability across diverse patient populations, and different countries and healthcare models, including low-resource settings and community-based rehabilitation services.

### Conclusion

This exploratory study presents a novel data-driven approach to establishing functional staging cutoff scores for the ClinFIT tool, utilizing a robust, ROC-based methodology grounded in real-world clinical data. The identified cutoff scores enable effective stratification of patients into light, moderate, and high rehabilitation intensity categories based on their total ClinFIT scores on admission. These thresholds demonstrated satisfactory discriminatory performance across the overall sample and key demographic and diagnostic subgroups, highlighting their potential utility in routine clinical decision-making.

The findings underscore ClinFIT’s capacity as a standardized assessment instrument and a practical tool for clinically meaningful triage and resource allocation. Integrating functional staging into routine clinical workflows may enable clinicians to prioritize rehabilitation interventions, align care intensity with patient needs, and facilitate coordinated interdisciplinary planning. This may contribute to improved patient outcomes and more efficient service delivery. It also has potential implications for policymakers and health planners seeking to optimize resource allocation and promote equitable access to rehabilitation. However, the proposed cutoffs should be interpreted as preliminary, and further external validation in larger, multicentre, and more diverse populations is essential to confirm their generalizability and reliability. Future research should focus on the prospective application of these thresholds in clinical practice, examining their predictive validity for outcomes such as discharge destination or length of stay, and exploring the feasibility of digital integration into EMR systems to facilitate real-time clinical application.

## Data Availability

The data associated with the manuscript are available from the corresponding author upon reasonable request. No AI tools or services were used during the preparation of this manuscript.
